# Intranasal administration of abatacept enhances IL-35^+^ and IL-10^+^ producing Bregs in lung tissues of ovalbumin-sensitized asthmatic mice model

**DOI:** 10.1371/journal.pone.0271689

**Published:** 2022-09-06

**Authors:** Maha Fahad Alenazy, Fatemeh Saheb Sharif-Askari, Mohammed S. El-Wetidy, Narjes Saheb Sharif-Askari, Ibrahim Yaseen Hachim, Mohammad-Hani Temsah, Basema Saddik, Roua Al-Kufaidy, Maha A. Omair, Yasser A. Alshawakir, Amany Adulgadel Fathaddin, Suad Hannawi, Qutayba Hamid, Mohammed A. Omair, Saleh Al-Muhsen, Rabih Halwani

**Affiliations:** 1 Department of Pediatrics, Immunology Research Lab, College of Medicine, King Saud University, Riyadh, Saudi Arabia; 2 Department of Physiology, College of Medicine, King Saud University, Riyadh, Saudi Arabia; 3 Sharjah Institute of Medical Research, College of Medicine, University of Sharjah, Sharjah, United Arab Emirates; 4 College of Medicine Research Center, King Saud University, Riyadh, Saudi Arabia; 5 Department of Clinical Sciences, College of Medicine, University of Sharjah, Sharjah, United Arab Emirates; 6 Department of Family and Community Medicine, College of Medicine, University of Sharjah, Sharjah, United Arab Emirates; 7 Prince Naif Center for Immunology Research and Asthma Research Chair, College of Medicine, King Saud University, Riyadh, Saudi Arabia; 8 Department of Statistics and Operations Research, College of Science, King Saud University, Riyadh, Saudi Arabia; 9 Experimental Surgery and Animal Lab, College of Medicine, King Saud University, Riyadh, Saudi Arabia; 10 Department of Pathology, College of Medicine, King Saud University, Riyadh, Saudi Arabia; 11 King Saud University Medical City, King Saud University, Riyadh, Saudi Arabia; 12 Department of Medicine, Ministry of Health and Prevention, Dubai, United Arab Emirates; 13 Meakins-Christie Laboratories, Research Institute of the McGill University Health Center, Montreal, Quebec, Canada; 14 Department of Medicine, Rheumatology Unit, College of Medicine, King Saud University, Riyadh, Saudi Arabia; 15 Department of Pediatrics, College of Medicine, King Saud University, Riyadh, Saudi Arabia; University of Torino, ITALY

## Abstract

**Backgrounds:**

Treating asthmatic rheumatoid arthritis patients with abatacept has been shown to associate with better control of asthma symptoms. However, the mechanism behind that is not investigated.

**Methods:**

Ovalbumin (OVA)- sensitized BALB/c female mice were treated intranasally (IN) or intraperitoneally (IP) with abatacept 4 hrs before the OVA challenge. The effects of abatacept IN or IP on the lungs and blood levels of Tregs and Bregs and their production of immunosuppressive cytokines, were determined using FACS analysis and ELISA assay.

**Results:**

Treating OVA- sensitized asthmatic mice model with abatacept, IN or IP, reduced lung inflammation. IN treatment with abatacept increased the frequency of IL-35 and IL-10 producing Bregs in the lung tissues to a higher level compared to IP treatment. Moreover, the frequency of lungs LAG3^+^ Tregs was significantly increased following treatment. This was also associated with a reduction in lung tissue and serum IL-17 levels of treated mice.

**Conclusions:**

These results suggest that abatacept by enhancing IL-35^+^IL-10^+^ Bregs and LAG3^+^ Tregs might reverse IL-17 induced lung inflammation during asthma.

## Introduction

The prevalence of chronic inflammatory diseases and asthma is increasing worldwide, which is a major challenge that threatens human health and a burden to economy. Moreover, the complexity and severity of asthma continue to increase especially in adults with systemic inflammatory diseases such as rheumatoid arthritis (RA) [1]. Asthma is characterized by airway hyperresponsiveness, mucus overproduction, and Th-2/Th-17 inflammatory type cell accumulation in the airways. The current most commonly used effective regimen is corticosteroid treatment, but many patients develop steroid hypo-responsiveness and progress to more severe disease stages. The development of new targeted therapeutic approaches, or repurposing biologics with better clinical efficacy and fewer side effects is imperative in improving treatment in steroid resistant patients. Several biologics are used for the treatment of interleukin (IL)-17 derived inflammation, especially in RA [2]. Recently, we showed that treatment of RA patients with abatacept for more than 6 months significantly lowered serum level of IL-17 [3]. While pathogenic Th-17 responses have been associated with asthma severity [4], abatacept ability to suppress these responses suggests their potential repurposing for the treatment of chronic respiratory diseases especially, at severe late stages of the disease.

Abatacept, a humanized version of CTLA-4Ig [5], was shown to be effective for the treatment of moderate to severe RA patients with an inadequate response to TNF-α therapy [6]. It competes with CD28 for CD80 (B7-1) or CD86 (B7-2) binding; and thereby, selectively ameliorate T-cell activation [7]. Besides its CD28 dependent T cell inhibitory mechanism, several reports have shown that abatacept enhances proliferation and suppressive function of regulatory T cells (Tregs) [8–10]. Treatment of CD4^+^ T cells isolated from RA patients with abatacept was shown to enhance the frequency of IL-10 producing LAG3^+^ Tregs [11], a potent subtype of Tregs known to produce high levels of IL-10 [12] and IL-35 [13]. These immunosuppressive mediators regulate the production of IL-10^+^ as well as IL-35^+^ producing regulatory B cells (Bregs), which are mostly CD138^+^ cells [12, 14–16]. Accordingly, we found that abatacept enhanced blood LAG3^+^ conventional (CD4^+^CD25^+^Foxp3^+^) and unconventional (CD4^+^CD25^-^Foxp3^+^) Tregs level as well as IL-35^+^IL-10^+^ producing Bregs (IL-35^+^IL-10^+^ cells within CD19^+^CD138^+^CD1d^+^) in RA patients. Interestingly, the blood level of IL-35^+^IL-10^+^ Bregs in remitted RA patients (DAS28-CRP <2.6) was significantly higher compared to that of unremitted [3]. Collectively, this could explain the enhanced differentiation of Tregs and Bregs following abatacept treatment. More work, however, is need to further characterize these mechanisms.

Relatively little is known about the effect of abatacept on inflamed lungs. In a clinical trial, abatacept was shown to be effective in RA with concurrent interstitial lung disease [17]. In an OVA-induced experimental mouse model of asthma which was developed through peripheral sensitization and airway challenge with ovalbumin, CTLA-4 intraperitoneal treatment reduced lung inflammation, and conversely, CTLA‐4Ig blockage led to development of eosinophilic lung infiltrates and BAL fluid eosinophilia [18]. Moreover, administration of cytoplasmic domain of CTLA-4 intranasally, reduced Th-2 cytokine levels such as IL-4, IL-5, and IL-13 in BAL fluid of OVA-sensitized mice [19]. Although these studies demonstrate the capacity for abatacept to dampen allergic inflammation in the lungs, further mechanistic work is required to determine the functional relevance of its local administration to the inflamed lung tissues.

Therefore, in this study we investigated the ability of abatacept to control lung inflammation and to enhance expansion of regulatory B and T cells in an OVA-sensitized asthmatic mice model. The effect of route of administration, intraperitoneally or intranasal, on abatacept efficiency to expand lungs and blood levels of Bregs and Tregs was also determined.

## Materials and methods

### Ethics statement

Mouse care and experimental procedures were performed following approval from Animal Care Committee of King Khalid University Hospital (KKUH). Mice were kept in sterile, individually ventilated-cages (TECNIPLAST) and fed a sterile, maintenance diet (Altromin 1324 TPF) and sterile-distilled water. Environmental enrichment was provided by autoclaved dust-free aspen bedding (ABEDD). For euthanasia at the end of the experiments, the mice were first anesthetized by isoflurane inhalation and then euthanized by cervical dislocation; all efforts were made to alleviate suffering.

### OVA-induced allergic inflammation model

We maintained 8-week-old female BALB/c (weighting 14.9 to 17.3 g) mice under pathogen-free conditions. The study consisted of 4 groups of mice: control, OVA challenged, OVA challenged mice treated with abatacept intranasally (OVA abatacept-IN), and OVA challenged mice treated with abatacept intraperitoneally (OVA abatacept-IP). In each group we had 10 mice which the lung tissues of 5 mice were used for histopathology and quantitative reverse transcriptase–polymerase chain reaction (qRT-PCR), and lung tissues of other 5 mice were used for flow cytometry. Peripheral blood mononuclear cells (PBMCs) were isolated from blood of all the 10 mice in each group and were used for the flow cytometry and the serum were used for the enzyme-linked immunosorbent assay (ELISA).

**Fig 1:**
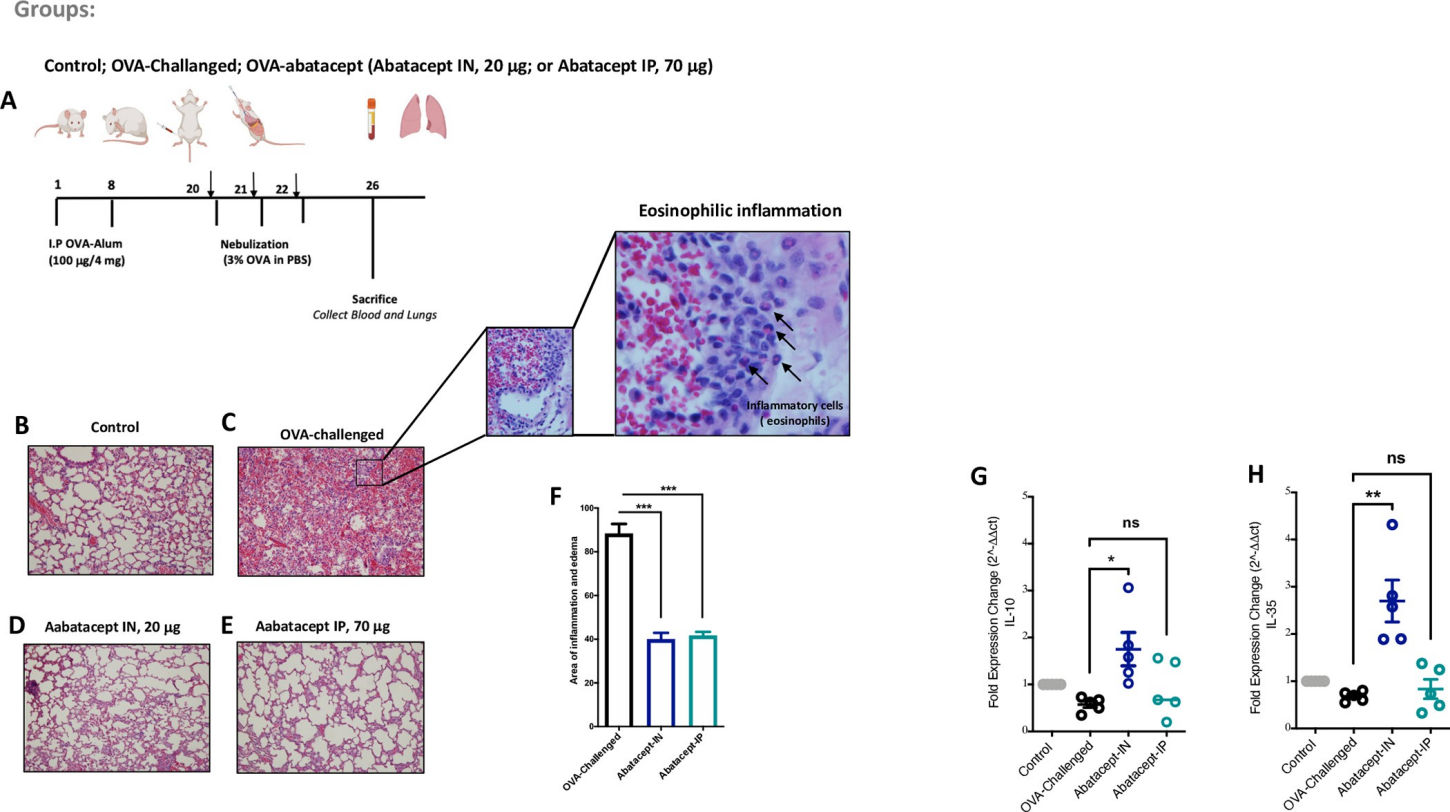
Abatacept attenuates acute lung tissues inflammation induced by OVA-challenge. (A) Schematic representation of the experimental procedure. Mice were sensitized with two intraperitoneal injections of ovalbumin (OVA) or PBS in an alum adjuvant at days 1 and 8. The mice received 20 μg of abatacept intranasally (IN), 70 μg of abatacept intraperitoneally (IP), or not, before the 3% OVA nebulizer challenge on the day 20, 21, and 22. (B-F) Lung sections were stained with haematoxylin and eosin (H&E) to measure the numbers of infiltrated inflammatory cells after the OVA-challenge, and with abatacept IN or with abatacept IP. Data shows that abatacept IN or IP attenuated acute lung tissues inflammation induced by OVA-challenge. (G and H) gene expression levels of IL-10 and IL-35 in the lung tissues of abatacept IN, abatacept IP treated, or OVA challenged mice. Data show an increase in IL-10 and IL-35 gene expression levels in the lung tissues following treatment with abatacept IN. n = 5 mice. Two-way comparison was done using t-test. ns: non-significant. * P<0.05, ** P<0.01, *** P<0.001.

We sensitized mice on day 1 and day 8 by intraperitoneal injection of 100 μg OVA (Sigma) emulsified in 4 mg aluminum hydroxide (Sigma). On days 20, 21 and 22 after the initial sensitization, we challenged mice with 3% OVA using a nebulizer (Fig 1). Each mouse received, or not, 20 μg of abatacept intranasally, 4 hours before the OVA challenge. The intranasal administration of abatacept was perfumed under inhalation anesthesia using isoflurane chamber. For the intraperitoneal treatment, each mouse received, or not, 70 μg of abatacept, 4 hours before the OVA challenge.

### Histopathological analyses

Lungs were obtained from the following groups of mice following the last instillation: control, OVA challenged, OVA abatacept-IN, and OVA abatacept-IP. The lung tissues were embedded in paraffin blocks which were cut in sets of four consecutive 5-μm-thick sections using an automatic microtome (SLEE Medical GmbH, Germany). Slides were stained with haematoxylin and eosin (H&E) to assess lung inflammation following OVA sensitization and the abatacept-IN or abatacept-IP treatments.

### Cell preparation and flow cytometry analysis

To isolated lung lymphocytes, mice lungs were collected in complete culture RPMI-1640 medium. Lung tissues were minced gently in complete medium, and the filtrates containing lung lymphocytes and other inflammatory cells were collected. The viability of the freshly isolated cells was evaluated by the Trypan blue dye exclusion test. PBMCs were isolated from blood samples using a Ficoll gradient (Axis Shield, Norway). PBMCs or lung cells were stained with APC anti-mouse CD19 antibody (BioLegend cat # 115512), APC/Cyanine7 anti-mouse CD138 (Syndecan-1) antibody (BioLegend cat # 142530), PerCP/Cyanine5.5 anti-mouse CD1d antibody (BioLegend cat # 123514), APC anti-mouse CD25 antibody (BioLegend cat # 102012), and PE anti-mouse CD223 (LAG-3) antibody (BioLegend cat # 125208). After surface staining, cells were stained with PE anti-mouse IL-27 p28 antibody (BioLegend cat # 516908), PE/Cy7 anti-mouse IL-10 antibody (BioLegend cat # 505026), and FITC anti-mouse FOXP3 antibody. Corresponding isotype controls were used for each antibody. All cells were analyzed with BD LSR II flow cytometer using DIVA software version 8.0. The corresponding data is deposited in S file.

### ELISA detection of serum IL-17

Serum cytokine IL-17 concentrations were determined using commercially available ELISA kit (BioLegend’s LEGEND MAX™ Mouse IL-17A ELISA Kit). Assays were performed strictly following the manufacturer’s instructions. All samples were measured in duplicates.

### Quantitative Polymerase Chain Reaction (qPCR)

Total RNA was extracted from the mice lung homogenate using RNeasy Mini Kit (Qiagen). For cDNA amplification 5x Hot FirePol EvaGreen qRT-PCR SuperMix (Solis Biodyne) was used and RT-qPCR was performed in QuantStudio 3 Real-Time PCR System (Applied Biosystems). The following primers were used: IL-35, forward, 5’-3’: CTCCTAGCCTTTGTGGCTGA, and reverse, 5’-3’: GCTCCAGTCACTTGGTTTCC; IL-10, forward, 5’-3’: CAGAGCCACATGCTCCTAGA, and reverse, 5’-3’: TCTCACCCAGGGAATTCAAA; GAPDH, forward, 5’-3’: TGTAGACCATGTAGTTGAGGTCA, and reverse, 5’-3’: AGGTCGGTGTGAACGGATTTG. Gene expression was analyzed using the Comparative Ct (ΔΔCt) method after normalization to the housekeeping gene GAPDH rRNA.

### Statistical analysis

Statistical comparisons were performed by using the independent samples *t*-test if the data were normally distributed, or the Mann–Whitney U-tests if the data were skewed. The Pearson correlation was used for correlating blood levels of IL-35^+^IL-10^+^ producing Breg as well as LAG3^+^cTreg and LAG3^+^uTreg with lung tissue and serum levels of IL-17. The analysis was performed using R software (v 3.0.2), SPSS Version 26 (IBM Corporation, Chicago, USA) and Graphpad Prism 7 (GraphPad Software Inc., San Diego, USA).

## Results

### Abatacept attenuated acute lung tissues inflammation induced by OVA-challenge

For the purpose of this study, we developed an OVA-sensitized mice model of acute lung inflammation [20]. Abatacept were delivered to mice intraperitoneally (IP), representing its standard subcutaneous route, or intranasally (IN), the pulmonary route for local lung treatment (Fig 1A). First, we assessed the degree of lung inflammation induced by OVA and following treatment with abatacept. As it is illustrated in Fig 1B–1F, 1H & 1E staining of lung tissue section showed eosinophilic infiltration in pulmonary vessels, alveolar ducts, and alveoli of OVA challenged lung tissue, and a significant reduction of inflammation and edema in OVA-challenged lung tissue following either IN or IP treatment with abatacept.

We further investigated the ability of IN and IP treatment with abatacept to induce IL-10 and IL-35 immune suppressive cytokines in blood and lung tissue of treated mice. Interestingly, we found that IN, but not IP, treatment with abatacept significantly elevated gene expression level of IL-10 and IL-35 in mice lungs (IL-10 increased by 1.7-folds; *P* = 0.011, and IL-35 increased by 2.7-folds; *P* = 0.002, in IN-treated compared to OVA-sensitized lung tissues) (Fig 1G and 1H).

### Abatacept enhances IL-35^+^ IL-10^+^ Breg levels in lung tissues and peripheral blood of OVA- sensitized mice

**Fig 2:**
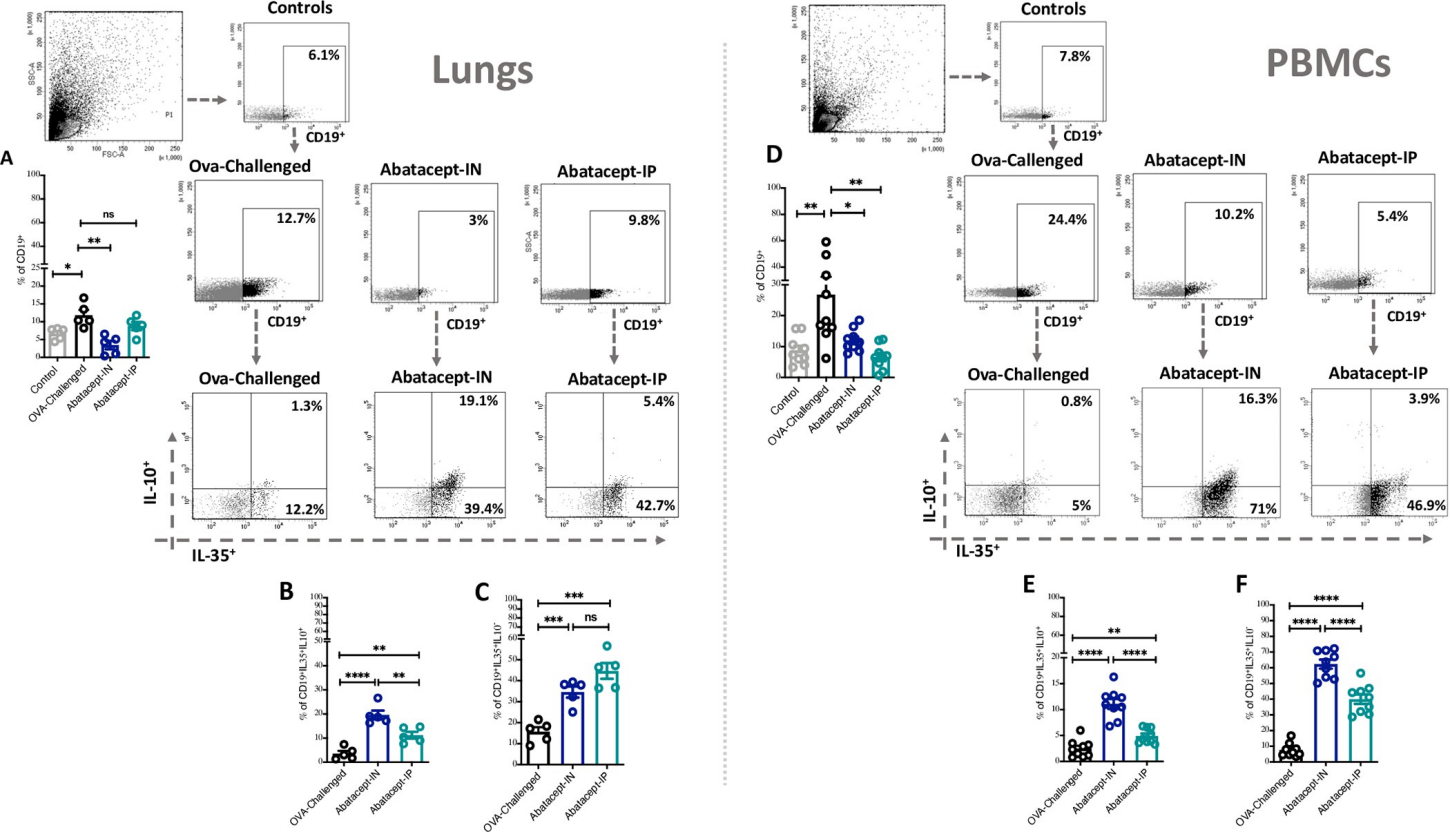
Abatacept enhances the levels of IL-35^+^IL-10^+^ and IL-35^+^ producing CD19^+^ cells in lung tissues and peripheral blood of OVA- sensitized mice. **(A)** Percentage of CD19^+^ cells in the lung tissues of abatacept IN, abatacept IP treated, or OVA challenged mice, and in the normal control mice. **(B-C)** Percentage of IL-35^+^IL-10^+^ producing B cells and IL-35^+^ producing B cells in the lung tissues of abatacept IN, abatacept IP treated, or OVA challenged mice. **(D)** Percentage of CD19^+^ cells in the PBMCs of abatacept IN, abatacept IP treated, or OVA challenged mice, and in the normal control mice. **(E-F)** Percentage of IL-35^+^IL-10^+^ producing B cells and IL-35^+^ producing B cells in the PBMCs of abatacept IN, abatacept IP treated, or OVA challenged mice. Lung tissues of 5 mice and PBMCs of 10 mice were used. Two-way unpaired t-test was used to calculate significance. * ***P < 0.001. Events were recorded and analysed by using BD FACSDiva version 8.0.

To evaluate the effect of abatacept on the level and function of IL-35^+^ IL-10^+^ producing regulatory B cells (Bregs) in inflamed lung tissues, we first examined the level of CD19^+^ B-cells in these tissues before and after IN and IP treatment with abatacept. As expected, the level of CD19^+^ cells in lung tissues (11.8% vs. 6.6% percentage of CD19^+^ cells in OVA-sensitized vs. control lung tissues, respectively; P = 0.014) as well as in peripheral blood (26.8% vs. 8.7% percentage of CD19^+^ cells in OVA-sensitized vs. control lung tissues, respectively; P = 0.008) of OVA-sensitized mice were elevated compared to the control group (Fig 2A and 2D). However, the frequency of CD19^+^ cells in lung tissues was significantly reduced following IN but not IP treatment with abatacept (7.6%, P = 0.002 and 2.2%, P = 0.145 decrease in percentage of CD19^+^ cells in IN-treated and IP-treated lung tissues, respectively) (Fig 2A). In contrast, in peripheral blood, this reduction in B cell counts was slightly more pronounced following IP treatment than IN treatment with abatacept (14.8%, P = 0.025 and 20.2%, P = 0.004 decrease in percentage of CD19^+^ cells in IN-treated and IP-treated PBMCs, respectively) (Fig 2D).

Next, we have investigated the effect of abatacept on the level and function of CD19^+^ B- cells that express immune suppressive cytokines IL-10 and IL-35. Interestingly, we found that IN treatment with abatacept significantly expanded the lungs and blood levels of CD19^+^IL-35^+^IL-10^+^ (Fig 2B and 2E) and CD19^+^IL-35^+^IL-10^-^ (Fig 2C and 2F). The increase in IL-35^+^IL-10^+^ B cells was higher following IN compared to IP abatacept treatment (19.5%, P<0.0001 and 11.2%, P = 0.002 percentage of IL-35^+^IL-10^+^ cells in IN-treated and IP-treated lung tissues, respectively; as well as 11.1%, P<0.0001 and 4.9%, P = 0.004, percentage of IL-35^+^IL-10^+^ cells in IN-treated and IP-treated PBMCs, respectively) (Fig 2B and 2E).

**Fig 3:**
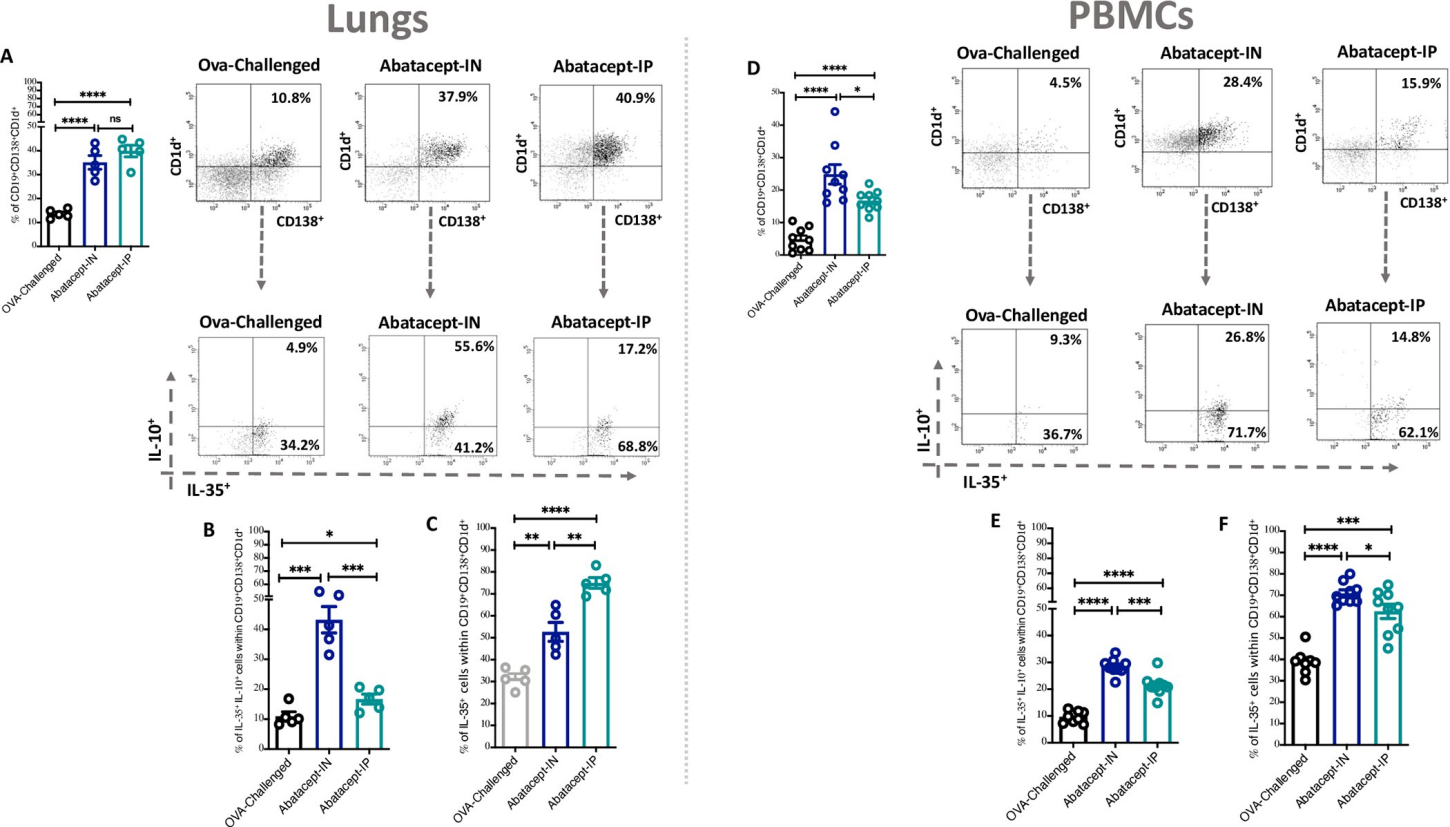
Abatacept enhances the levels of IL-35 and IL-10 producing Bregs in lung tissues and peripheral blood of OVA- sensitized mice. **(A)** Percentage of CD19^+^CD138^+^CD1d^+^ cells in the lung tissues of abatacept IN, abatacept IP treated, or OVA challenged mice. **(B-C)** Percentage of IL-35^+^IL-10^+^ Bregs and IL-35^+^ Bregs in the lung tissues of abatacept IN, abatacept IP treated, or OVA challenged mice. **(D)** Percentage of CD19^+^CD138^+^CD1d^+^ cells in the PBMCs of abatacept IN, abatacept IP treated, or OVA challenged mice. **(E-F)** Percentage of IL-35^+^IL-10^+^ Bregs and IL-35^+^ Bregs in the PBMCs of abatacept IN, abatacept IP treated, or OVA challenged mice. Lung tissues of 5 mice and PBMCs of 10 mice were used. Two-way unpaired t-test was used to calculate significance. * ***P < 0.001. Events were recorded and analysed by using BD FACSDiva version 8.0.

We then assessed the effect of abatacept on the lung tissues as well as blood level of CD19^+^CD138^+^CD1d^+^ Breg subtype and found an increase in this Breg with either IN or IP abatacept treatment (Fig 3A and 3D). Furthermore, we have evaluated the level of IL-35^+^IL-10^+^ Breg (% of IL-35^+^IL-10^+^ cells within CD19^+^CD138^+^CD1d^+^) as well as IL-35^+^ Breg (% of IL-35^+^ cells within CD19^+^CD138^+^CD1d^+^) in the lung tissues as well as blood levels following IN or IP treatment with abatacept. Although both routes of abatacept administration enhanced the level of these Breg subsets in both lung tissues and peripheral blood (Fig 3B, 3C, 3E, and 3F); IN treatment was much more efficient in enhancing IL-35^+^IL-10^+^ Breg to significantly higher levels compared to IP treatment in lung tissues (43.2%, P = 0.0001 and 16.7%, P = 0.033 percentage of IL-35^+^IL-10^+^ cells in IN-treated and IP-treated lung tissues) (Fig 3B).

### Abatacept intranasally enhanced LAG3^+^ cTreg and LAG3^+^ uTreg levels in lung tissues and peripheral blood of OVA-sensitized mice

**Fig 4:**
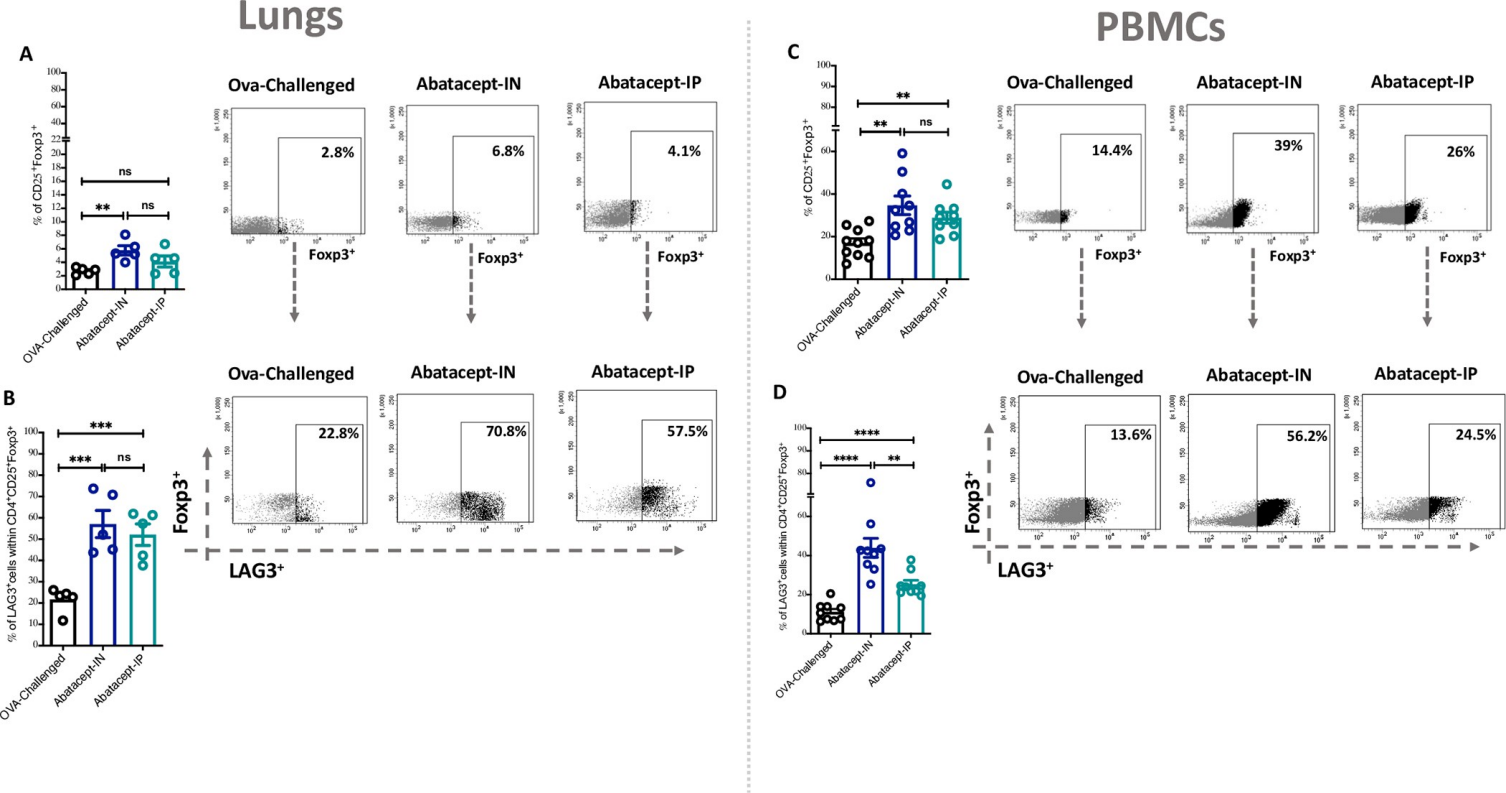
Abatacept enhances the levels of conventional Tregs (cTregs) in lung tissues and peripheral blood of OVA- sensitized mice. **(A)** Percentage of cTregs (Foxp3^+^ cells within CD4^+^CD25^+^) in the lung tissues of abatacept IN, abatacept IP treated, or OVA challenged mice. **(B)** Percentage of LAG3^+^ cTregs (CD25^+^Foxp3^+^LAG3^+^) in the lung tissues of abatacept IN, abatacept IP treated, or OVA challenged mice. **(C)** Percentage of cTregs in the PBMCs of abatacept IN, abatacept IP treated, or OVA challenged mice. **(D)** Percentage of LAG3^+^ cTregs in the lung tissues of abatacept IN, abatacept IP treated, or OVA challenged mice. Lung tissues of 5 mice and PBMCs of 10 mice were used. Two-way unpaired t-test was used to calculate significance. * ***P < 0.001. Events were recorded and analysed by using BD FACSDiva version 8.0.

**Fig 5:**
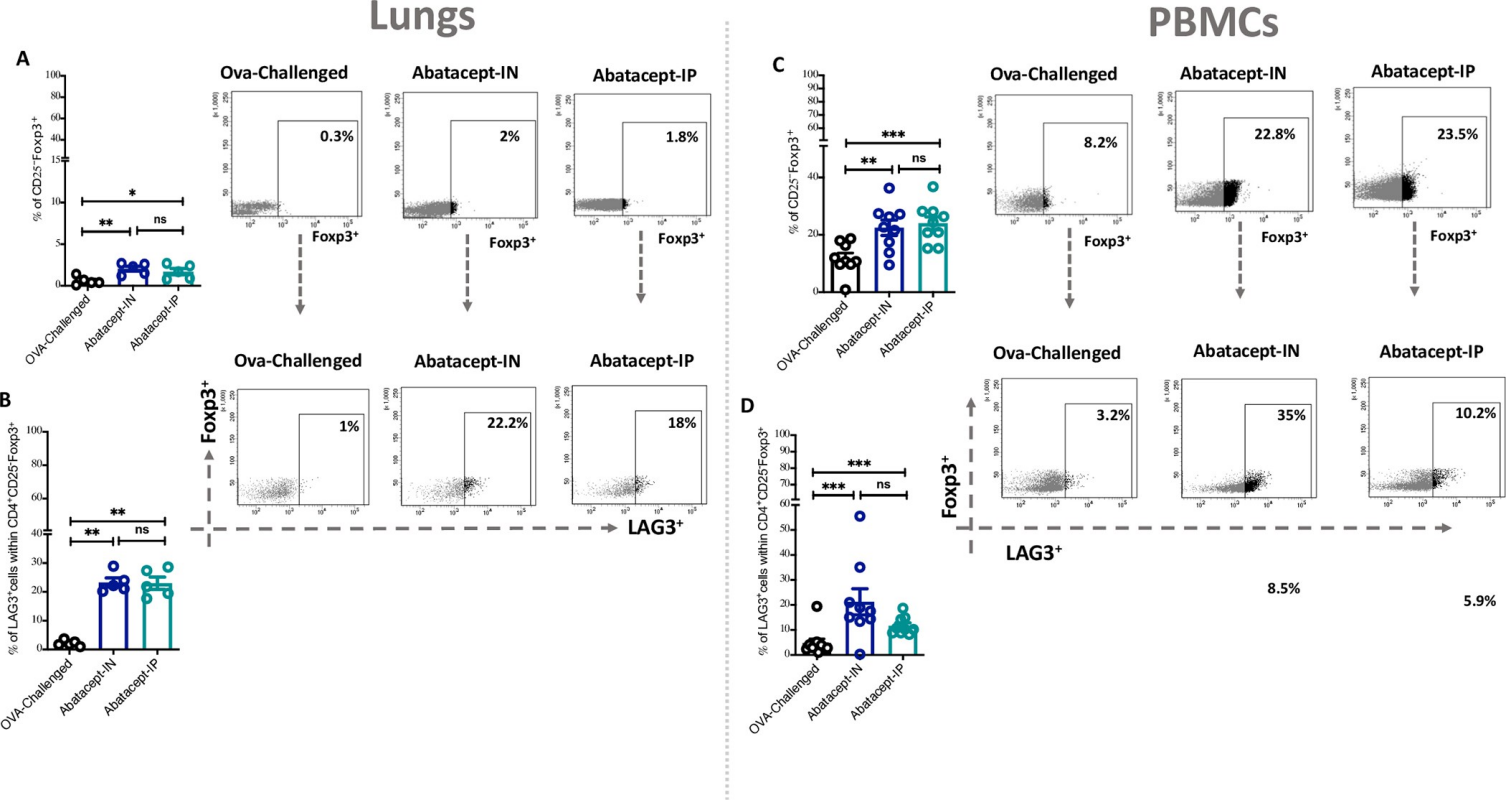
Abatacept enhances the levels of unconventional Tregs (uTregs) in lung tissues and peripheral blood of OVA- sensitized mice. **(A)** Percentage of uTregs (Foxp3^+^ cells within CD4^+^CD25^-^) in the lung tissues of abatacept IN, abatacept IP treated, or OVA challenged mice. **(B)** Percentage of LAG3^+^ uTregs (CD25^-^Foxp3^+^LAG3^+^) in the lung tissues of abatacept IN, abatacept IP treated, or OVA challenged mice. **(C)** Percentage of uTregs in the PBMCs of abatacept IN, abatacept IP treated, or OVA challenged mice. **(D)** Percentage of LAG3^+^ uTregs in the lung tissues of abatacept IN, abatacept IP treated, or OVA challenged mice. Lung tissues of 5 mice and PBMCs of 10 mice were used. Two-way unpaired t-test was used to calculate significance. ****P < 0.001. Events were recorded and analysed by using BD FACSDiva version 8.0.

Next, since the effect of abatacept on Treg cells within inflamed lung tissues remains unknown, we have evaluated the effect of IN or IP treatment with abatacept on lung tissue as well as blood levels of conventional Tregs (cTregs; CD25^+^Foxp3^+^) and unconventional Tregs (uTregs; CD25^-^Foxp3^+^). Of interest, IN and IP treatment with abatacept enhanced the levels of cTregs and uTregs in lung tissues and peripheral blood of treated mice, however, this effect was more pronounced with the IN treatment for enhancing the level of cTregs (5.7%, P = 0.002 and 4.1%, P = 0.137 percentage of cTregs in IN-treated and IP-treated lung tissues, respectively; as well as 34.6%, P = 0.001 and 28.8%, P = 0.002, percentage of cTregs in IN-treated and IP-treated PBMCs, respectively)

(Fig 4A and 4C; for cTregs, and Fig 5A and 5C for uTregs level in lung tissues and PBMCs, respectively). Notably, both IN and IP treatments expanded the levels of LAG3^+^ cTreg and LAG3^+^ uTreg in lung tissues and peripheral blood of treated mice, however, the effect was more pronounced with the IN treatment for increasing the level of cTregs (57.1%, P = 0.0009 and 52.1%, P = 0.0007 percentage of cTregs in IN-treated and IP-treated lung tissues, respectively; as well as 43.9%, P<0.0001 and 25.3%, P<0.0001, percentage of cTregs in IN-treated and IP-treated PBMCs, respectively) (Fig 4B and 4D, for lung and blood LAG3^+^ cTreg; and Fig 5C and 5D for lung and blood LAG3+ uTreg, respectively).

### Intranasal abatacept reduced lung tissue and serum IL-17 level of OVA- sensitized mice

**Fig 6:**
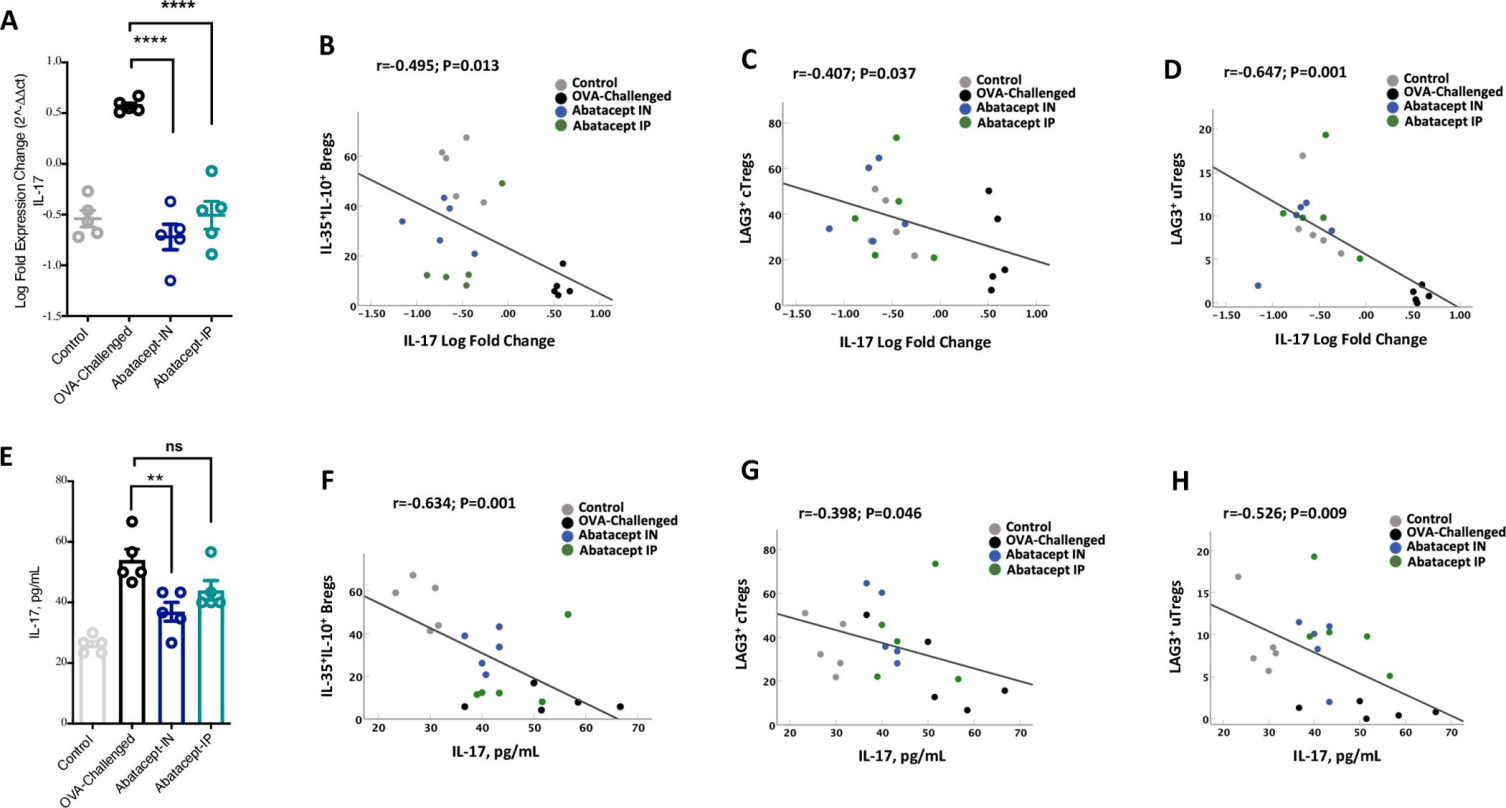
Lung tissue and serum levels of IL-17 are suppressed following abatacept treatment and negatively correlated with blood Breg and Treg levels. **(A)** Lung tissue gene expression level of IL-17 following treatment with the abatacept IN and IP. **(B-D)** Correlation between percentage of IL-135^+^IL-10^+^ producing Bregs, LAG3^+^ cTregs, or LAG3^+^ uTregs in the lung and lung level of IL-17. **(E)** Serum level of IL-17 following treatment with the abatacept IN or IP. **(F-H)** Correlation between percentage of IL-135^+^IL-10^+^ producing Bregs, LAG3^+^ cTregs, or LAG3^+^ uTregs in the lung and serum level of IL-17. Lung tissues of 5 mice and PBMCs of 10 mice were used. Statistical test; Pearson’s correlation coefficient with a one‐sided test for significance (P<0.05 significant).

In this study we have also determined whether abatacept treatment suppresses lung tissue inflammation by downregulating IL-17 levels. Interestingly, we found lower lung tissue gene expression level of IL-17 with IN abatacept and IP abatacept treatment (IL-17 decreased by 1.3 Log folds by IN abatacept, P<0.001; and about a Log fold decreased by IP abatacept treatment compared to OVA-challenged lung, P<0.001). Moreover, lung IL-17 level was negatively correlated with lung tissue levels of IL-35^+^IL-10^+^ producing Breg (Fig 6B; r = -0.495, P = 0.013), LAG3^+^cTreg (Fig 6C; r = -0.407, P = 0.037), and LAG3^+^uTreg (Fig 6D; r = -0.647, P = 0.001).

Additionally, lower serum level of IL-17 was associated with IN treatment with abatacept to a higher level compared to IP treatment (36.9 pg/mL; P = 0.006, and 43.3 pg/mL; P = 0.071, IL-17 level in serum of IN-treated and IP-treated mice compared to OVA-challenged mice 55 pg/mL, respectively) (Fig 6E). Likewise, to the lung IL-17 level, serum IL-17 level was negatively correlated with lung tissue levels of IL-35^+^IL-10^+^ producing Breg (Fig 6F; r = -0.634, P = 0.001), LAG3^+^cTreg (r = -0.398, P = 0.046), and LAG3^+^uTreg (r = -0.526, P = 0.009). These results highlight the molecular mechanisms regulating the unique immune-suppressing effect of abatacept to lung tissue inflammation.

## Discussion

In this study we have shown that lung inflammation was significantly reduced, as well as the frequency of IL-35^+^IL-10^+^ producing Breg was enhanced in the lung tissue of OVA-sensitized mice following local intranasal treatment with abatacept, and to a higher level compared to its systemic intraperitoneal administration. IL-17 lung and serum levels were also significantly reduced in these mice. These results indicated that abatacept could be repurposed for the treatment of severe asthma as it is able to attenuate the pathogenic role of IL-17 in asthma.

The known mode of action of abatacept is through inhibition of CD28-mediated costimulation. Inhibition of this pathway with CTLA4Ig has been shown to potently inhibit allergic airway inflammation in asthma animal models. However, we show for the first time that abatacept induces the expansion of CD138^+^CD1d^+^ Bregs producing IL-35^+^ and IL-10^+^ cytokines in the inflamed lung tissue of OVA-challenged mice. The fact that CD138^hi^ plasma cells are the major source of IL-35^+^ and IL-10^+^ B cells have been highlighted in mice model of Experimental Autoimmune Encephalomyelitis (EAE) and *Salmonella* infection [16]. CD138^hi^ plasma cells isolated from these mice expressed significantly higher levels of IL-35 and IL-10 compared to CD19^+^CD138^-^ B cells [16]. Moreover, EAE mice deficient in IL-35 producing B cells (B-p35^-\-^) displayed increased IL-17 production compared to control mice. B-p35^-\-^ mice also had more CD4^+^ T cells in the central nervous system than wild type B cells mice, suggesting that B cell-derived IL-35^+^ is a critical regulator of CD4^+^ T-cell mediated immunity and Th17 responses [16]. Likewise, a recent study demonstrated that IL-35 is critical in suppressing superantigenic *Sataphylococcus aureus*-driven Th-17 responses in the upper respiratory tract [21] Treatment of experimental autoimmune uveitis (EAU) with recombinant IL-35 was shown to suppress Th-17 responses through expansion of IL-35^+^ and IL-10^+^ B cells [14]. It is also worth noting that in our study, lung levels of IL-35^+^IL-10^+^ Breg were negatively correlated with both lung and IL-17 serum levels of abatacept treated mice (Fig 6D). Future studies however are needed to assess whether regulatory B cells producing IL-10 and IL-35 could provide novel opportunities for immune regulation of lung inflammation especially during severe uncontrolled asthma.

In this study we have also shown that abatacept enhances the lung levels of LAG3^+^ cTregs and LAG3^+^ uTregs. LAG3, an MHC-class-II-binding CD4^+^ homologue [22] has been identified to suppress airway inflammation in a mouse model of OVA-induced allergic asthma [23]. The ability of LAG3^+^ Treg to control inflammation in the intestine was reported to be through IL-10/STAT3 axis and reduction of IL-17 secretion from the colon tissue culture [24]. STAT3 signaling in Tregs is a main pathway that allows Tregs to specifically suppress Th17 mediated responses [25]. IL-10 was also found to be a major inducer of STAT3 [24, 26], as IL-10RA^−/−^ Tregs mice spontaneously developed dysregulated Th17 type inflammation in the intestine, while other STAT3-activating cytokines, such as IL-6, were unable to exert the same effect [24]. Of note, lung IL-17 level was negatively correlated with LAG3^+^cTreg or LAG3^+^uTreg in the lung tissues of abatacept IN treated mice. Since IL-17 is a known marker of lung inflammation and asthma severity [4, 27], these data could imply the importance of expanding lung levels of LAG3^+^ Treg in asthmatic lung as the levels of LAG3^+^cTreg or LAG3^+^uTreg on the inflamed lung tissues were both correlated with lower serum level of IL-17.

The mechanism by which abatacept enhances Tregs differentiation and activation, however, is not fully understood. Binding of abatacept to B7 proteins is believed to trigger dendritic cells to express indoleamine 2,3 dioxygenase, a tryptophan-catabolizing enzyme that induces and activates CD4^+^CD25^+^ Treg [28, 29]. Abatacept or CTLA‐4Ig was also shown to inhibit Th17 cells and enhance Tregs activity through a transforming growth factor-beta (TGF-β) dependent mechanism [30]. Moreover, other than functional antagonism between Th17 and Treg cells, these cells can be generated under distinct cytokine conditions depending on the presence of TGF-β or TGF-β plus IL-6. In the absence of any inflammatory insult or IL-6, TGF-β induces Foxp3^+^ Tregs and suppresses generation of Th17 cells; and on infection or inflammation, IL-6 will suppress the generation of TGF-β-induced Treg cells and induce Th17 mediated response [31]. Th17 cells have been implicated in the pathogenesis of asthma due to their expression of IL-17 [32]. In lungs of asthma patients, the level of IL-17 correlates with asthma severity [4] and thus, the balance between Th17 cells and Treg is more shifted towards an increased number of Th17 cells with less functional Tregs. Moreover, expression of glucocorticoid receptor (GR) on Foxp3^+^ Tregs cells was found to be essential for glucocorticoid-mediated suppression of autoimmune inflammation in EAE mice, suggesting that glucocorticoid may directly acts on Tregs [33]. Therefore, restoring the balance and efficacy of Tregs may be important in the treatment of steroid resistance severe asthma.

In addition, abatacept has been approved for treatment of RA, providing the opportunity to test if it is effective to treat asthma. However, in contrast to the results from animal models, abatacept had contraindicatory results when it was given to eosinophilic asthma patients [34, 35]. Asthma is a heterogenous disease with different subsets (Th2-high or eosinophilic, Th17-high or neutrophilic, and Th2/Th17-low asthma) and responses to therapeutics. Here, we are proposing that abatacept administered locally to the lung is able to attenuate Th17-mediated inflammation during asthma by increasing level of IL-35 and Tregs [36]. Th17 cells contribute to steroid-resistant airway inflammation and airway hyperresponsiveness in mice model [32], and hence abatacept, by targeting IL-17 signaling, could improve responsiveness to steroid therapy in these steroid hypo-responsive patients.

In conclusion our study is the first to show that abatacept therapeutic effects extend toward enhancing IL-35 and IL-10 producing Breg subtype and LAG3^+^ cTregs or LAG3^+^ uTregs in the inflamed lung tissues. The effect on the lung tissues was more pronounced with the pulmonary route compared to the systemic route of abatacept administration.

## Supporting information

S1 File Study raw data. (CSV)
